# Selection and inheritance of sexually dimorphic juvenile plumage coloration

**DOI:** 10.1002/ece3.1793

**Published:** 2015-11-02

**Authors:** Angela Tringali, Reed Bowman, Arild Husby

**Affiliations:** ^1^ Avian Ecology Laboratory Archbold Biological Station 123 Main Dr. Venus Florida 33960; ^2^ Department of Biosciences University of Helsinki PO Box 65 FI‐00014 Helsinki Finland

**Keywords:** *Aphelocoma coerulescens*, intersexual genetic correlation, maternal effects, plumage color, sex linkage, Z‐linkage

## Abstract

Sexually dimorphic plumage coloration is widespread in birds and is generally thought to be a result of sexual selection for more ornamented males. Although many studies find an association between coloration and fitness related traits, few of these simultaneously examine selection and inheritance. Theory predicts that sex‐linked genetic variation can facilitate the evolution of dimorphism, and some empirical work supports this, but we still know very little about the extent of sex linkage of sexually dimorphic traits. We used a longitudinal study on juvenile Florida scrub‐jays (*Aphelocoma coerulescens*) to estimate strength of selection and autosomal and Z‐linked heritability of mean brightness, UV chroma, and hue. Although plumage coloration signals dominance in juveniles, there was no indication that plumage coloration was related to whether or not an individual bred or its lifetime reproductive success. While mean brightness and UV chroma are moderately heritable, hue is not. There was no evidence for sex‐linked inheritance of any trait with most of the variation explained by maternal effects. The genetic correlation between the sexes was high and not significantly different from unity. These results indicate that evolution of sexual dimorphism in this species is constrained by low sex‐linked heritability and high intersexual genetic correlation.

## Introduction

Sexually dimorphic coloration occurs in several taxa, and numerous studies have demonstrated the selective advantages of conspicuous coloration for mating success (Andersson 1994) and dominance‐status signaling (Bradbury and Davies 1987; Berglund et al. 1996; Senar 2006). Sexually dimorphic coloration is particularly widespread and well studied in birds. Many studies report correlations between plumage color and fitness related traits, including body condition (Siefferman and Hill 2005), mate choice (Hill 1990), and measures of fecundity (Badyaev et al. 2001; Safran and McGraw 2004; Siefferman and Hill 2005). Although genetic studies of color variation have increased substantially in the last decade (Fitze et al. 2003; Johnsen et al. 2003; Hadfield et al. 2006; Quesada and Senar 2009; Potti and Canal 2011; Husby et al. 2013; Roulin and Ducrest 2013; Vergara et al. 2015), few of these consider selection (but see McGlothlin et al. 2005; Vergara et al. 2015). To understand the evolution of sexually dimorphic coloration, we must quantify both selection and heritability.

Most of the heritability estimates of plumage coloration assume a strict autosomal genetic basis, a surprising assumption because sex‐linked inheritance of color was documented as early as 1927 in guppies (P*oecilia reticulata*) (Winge 1927). In guppies, this finding has subsequently been confirmed both by quantitative genetic (Houde 1992) and QTL studies (Tripathi et al. 2009) and is consistent with the idea that the evolution of sexual dimorphism (SD) would be facilitated by the genes for sexually dimorphic traits being located on the sex chromosomes (Rice 1984). Although sex‐linked inheritance of color has been documented in other taxa (Ellers and Boggs 2002; Miura et al. 2011), relatively few studies of the genetics of color in birds consider sex linkage (Roulin et al. 2010; Husby et al. 2013; Evans et al. 2014; Roulin and Jensen 2015).

Birds are a particularly interesting group in which to study the nonautosomal sources of variation because Z‐linkage of female preference facilitates the evolution of conspicuous male secondary sexual traits under Fisher's runaway model of sexual selection (Kirkpatrick and Hall 2004). Moreover, birds lack a global dosage compensation mechanism (Ellegren et al. 2007; Itoh et al. 2007; Arnold et al. 2008; Wolf and Bryk 2011), and thus, quantitative genetic methods are well suited to partition autosomal and sex chromosome‐linked genetic variance (Husby et al. 2013). However, because dosage compensation occurs on a gene‐by‐gene basis, information about gene‐specific dosage compensation along with gene‐specific relatedness matrices would be needed for a complete description of the role sex chromosomes have on phenotypic variation (Husby et al. 2013).

The Florida scrub‐jay (*Aphelocoma coerulescens*) is a suitable model organism to study the selection and inheritance of plumage coloration. Both sexually immature juvenile (Siefferman et al. 2008) and adult (Bridge et al. 2008) Florida scrub‐jays exhibit sexually dimorphic ultraviolet plumage coloration. In juveniles, this color is a signal of dominance status (Tringali and Bowman 2012), but its function in adults is unknown. Dominant individuals gain priority access to resources (Drews 1993), which may include food and breeding opportunities. Florida scrub‐jays are despotic cooperative breeders with limited opportunities to acquire breeding territories. When individuals do acquire breeding territories, it is near their natal territory, with dispersal distances of one territory length being most common (Woolfenden and Fitzpatrick 1984). Because of these short dispersal distances, juveniles are likely to interact with the same set of similar‐aged individuals throughout their lifetimes. Thus, establishing social dominance early may provide benefits later when competing for territories. For this reason, we predict that juvenile plumage will be under selection.

Here, we use data from an ongoing long‐term demographic study to quantify the genetic architecture of plumage color of juvenile Florida scrub‐jays. First, we examine whether plumage coloration is under selection in this population, as is suggested by the relationship between coloration and juvenile dominance status (Tringali and Bowman 2012). Then, to test the hypothesis that the evolution of SD is facilitated by sex‐linked inheritance or weak intersexual genetic correlation, we compare estimates of heritability assuming autosomal and sex‐linked inheritance and estimate the cross‐sex genetic correlation for plumage traits.

## Methods

### Study organism

Florida scrub‐jays are a territorial, nonmigratory, socially and genetically monogamous cooperative breeding species (Woolfenden and Fitzpatrick 1984; Townsend et al. 2011). These characteristics allow us to follow individuals for the duration of their lives and accurately determine parentage from field observations. Territories are usually held for life, and a pair nests on the same territory every year (Woolfenden and Fitzpatrick 1984). If one member of the pair is widowed, the other may remain on the territory and breed with a new mate or may settle on a new territory with a new mate.

Many adults never breed, and among breeders reproductive skew is high; most (51%) breeding males produce no breeding offspring over their lifetime and less than 10% produce five or more. Florida scrub‐jays disperse short distances and males frequently inherit all or part of their father's territory (Woolfenden and Fitzpatrick 1984). High reproductive skew coupled with short dispersal distance creates a pattern where highly successful lineages are often spatially clustered within the landscape.

### Study population and data collection

Our work was conducted on the population of Florida scrub‐jays long studied at Archbold Biological Station, Highlands County, FL (21**°**10′N, 81**°**21′W) (Woolfenden and Fitzpatrick 1984). All individuals in the population are marked with a unique combination of color bands, and each is monitored throughout its lifetime on the study area. Each year all nests are found, monitored, and their locations recorded with GPS.

Nestlings are banded with a single color band, and a blood sample is collected for genetic sex determination. These individuals are recaptured as juveniles, at approximately 65 d postfledging, at which time they are given a unique set of color bands and (since 1990) the outermost right tail feather is collected. Feathers are stored in individually labeled envelopes in a museum cabinet in a climate‐controlled room. UV‐blue coloration does not fade over the timespans that our feather samples were in storage (Armenta et al. 2008; Siefferman et al. 2008). We used this historical collection of feathers in our analyses.

To measure feather reflectance, we used an Ocean Optics USB‐4000 spectrometer (Ocean Optics, Dunedin, FL) connected to a DH‐200 deuterium halogen light source by a bifurcated fiber optic probe. We measured three 3.14 mm^2^ points on each feather, 1, 2, and 3 mm from the distal tip by holding the probe at a 90**°** angle 0.5 cm from the surface of the feather. We averaged these three measurements for each sample and then calculated mean brightness $\left(\sum R_{\lambda_{300-700}}/n_{w}\right)$, UV chroma $\left(\sum R_{\lambda_{300-400}}/\sum R_{\lambda_{300-700}}\right)$, and hue $\left(\lambda_{R_{\max}}\right)$, where *R* is reflectance, *λ* is wavelength, and *n*_w_ is number of wavelengths measured (Montgomerie 2006). We had a total of 3534 measurements from 1178 individuals (three different color measurements per individual, no repeated observations on the same individual, see Table 1).

**Table 1:** Least squares means ± standard error of color traits in juvenile male and female Florida scrub‐jays

Sex	Mean brightness	UV chroma	Hue
Female	9.326 ± 0.226	0.282 ± 0.007	396.822 ± 6.458
Male	9.039 ± 0.226	0.289 ± 0.007	388.626 ± 6.456
Sexual dimorphism	1.032	−1.025	1.021

Males and females differ significantly (*P* < 0.0001) in all measures of reflectance, but sexual dimorphism is slight.

### Sexual dimorphism

While previous studies of Florida scrub‐jay color have demonstrated SD (Bridge et al. 2008; Siefferman et al. 2008; Tringali and Bowman 2012), we re‐examined these results here as the sample size in our study is considerably larger than the previous studies. Following Lovich and Gibbons (1992), we calculated the degree of SD using the ratio of the trait mean of the sex (*μ*) with the larger value to the trait mean of the sex with the smaller value so that SD = *μ*_larger/_ *μ*_smaller_. The ratio is assigned a positive value if females had the larger trait value and negative if males had the larger value.

### Selection analyses

The importance of plumage coloration for social dominance among juveniles has been well established in this system (Tringali and Bowman 2012), but the strength of selection has not been estimated. Therefore, we used information on whether or not individuals bred and lifetime reproductive success (LRS) to calculate nonlinear selection gradients (Brodie et al. 1995) using generalized additive models (GAMs). We classified individuals as breeders if they produced at least one egg and measured LRS as the number of breeding offspring produced. Whether or not an individual bred was modeled as a binomial variable and LRS was modeled using a negative binomial distribution because it is strongly skewed. We limited these analysis to the 2005 and earlier cohorts to exclude birds who still have several reproductive years ahead of them. Of the 602 individuals included in this analysis, only 16 were still alive at the time of analysis. Color traits were standardized to create *z*‐scores. We used generalized linear models (GLMs) to estimate whether any metric of plumage reflectance predicted whether or not a bird bred. For LRS, we used the R‐package “mgcv” to run the GAMs (Wood 2011), with the color variables as covariates. Because additive models are by definition additive, we fit sex using the “by” function and compared these models to a model that did not include sex using AIC. The GAM estimates of selection were then converted to standardized selection gradients using the R‐package “GSG” (Morrissey and Sakrejda 2013). For color variables where the model that included sex had the lowest AIC, we fit separate selection gradients for males and females; otherwise, both sexes were fit together. Additionally, we used GLMs to estimate yearly standardized selection gradients (Lande and Arnold 1983).

### Pedigree information

We obtained information on coefficients of relatedness between individuals in this study from a pedigree reconstructed based on field observations. Because Florida scrub‐jays are both behaviorally and genetically monogamous (Townsend et al. 2011), the social pedigree reflects true genetic lineages. In total, the pedigree contained 1401 individuals, of which 249 maternities, 250 paternities, 2016 full‐sibling links, and 3866 half‐sibling links were informative for analysis of coloration. Mean relatedness was 0.0054 and was estimated using the R‐package pedantics (Morrissey and Wilson 2010).

### Quantitative genetic analyses

To partition variation in mean brightness, UV chroma, and hue, we used the above pedigree in a mixed model framework (Kruuk 2004). We first estimated the autosomal additive genetic basis of coloration (mean brightness, UV chroma, and hue) using the model:

(1) $$\text{Trait}=\text{Sex}+V_{A}+V_{M}+V_{T}+V_{R}$$

where trait refers to either mean brightness, UV chroma, or hue, sex is a two‐level factor to account for the slight SD in coloration (Siefferman et al. 2008), and *V*_A_ is the additive genetic autosomal variance, *V*_M_ is the variance associated with maternal identity, *V*_T_ is the variance due to territory identity, and *V*_R_ is the residual variance.

Second, we estimated the Z‐linked genetic relatedness matrix to estimate the proportion of genetic variance located on the macro sex chromosome. The full details can be found in Husby et al. (2013). Briefly, we expanded on the model above such that variation in the color traits was modeled as:

(2) $$\text{Trait}=\text{Sex}+V_{A}+V_{M}+V_{T}+V_{Z}+V_{R}$$

where *V*_Z_ represents sex‐linked genetic variance.

Sex‐linked genetic variance can be separated from the autosomal genetic variance because the Z‐linked and autosomal relatedness coefficients differ between some types of relatives (Grossman and Eisen 1989). Because females are the heterogametic sex (ZW) in birds, male offspring inherit one of their two Z chromosomes directly from their mother and female offspring their single Z chromosome from their father. As a result, the relatedness coefficient between two male full siblings, for example, will be 0.75 for any Z‐linked gene, compared to 0.5 for an autosomal gene. Some types of relatives have identical relatedness for both Z‐linked and autosomal markers (e.g., father–son relationship), and thus, the power to detect Z‐linked genetic variance is lower than for autosomal genetic variance (Husby et al. 2013).

### Multivariate quantitative genetic models

Blue and ultraviolet plumage coloration is structural (Prum 2006), and therefore, it is possible that the color parameters we measured are not independent of each other. To examine this possibility, we ran multivariate models to test for phenotypic and genetic dependencies between the color traits. Note that for the genetic model we only examined UV chroma and brightness because we could not detect any additive genetic basis to hue (Table 2); hence, a genetic correlation is not defined. Our bivariate phenotypic model was therefore:

(3) $$\text{Trait}1\;\text{Trait}2=\text{Sex}+V_{I}+V_{M}+V_{T}+V_{R}$$

where *V*_I_ is the between‐individual variance. We extended this model to a bivariate animal model as:

(4) $$\text{UV}\;\text{chroma brightness}=\text{Sex}+V_{A}+V_{M}+V_{T}+V_{R}$$

**Table 2:** Yearly standardized selection gradients for three components of plumage reflectance in Florida scrub‐jays juveniles

Year	Mean brightness			UV chroma			Hue		
	Slope	SE	*P* value	Slope	SE	*P* value	Slope	SE	*P* value
1990	−0.447	1.390	0.802	−1.107	0.882	0.428	−0.413	0.546	0.587
1991	−0.043	0.122	0.725	0.050	0.123	0.689	0.040	0.121	0.744
1992	−0.294	0.233	0.247	0.128	0.292	0.674	−0.206	0.365	0.590
1993	−0.744	0.206	**0.001**	−0.067	0.230	0.773	0.192	0.218	0.385
1997	0.087	0.117	0.487	0.055	0.141	0.713	−0.062	0.131	0.658
1998	−0.602	0.349	0.108	−0.350	0.353	0.340	0.210	0.276	0.461
1999	0.255	0.154	0.107	−0.123	0.178	0.494	0.055	0.177	0.758
2000	−0.097	0.196	0.625	−0.196	0.287	0.504	0.221	0.271	0.424
2001	0.324	0.228	0.178	0.917	0.634	0.171	−0.143	0.281	0.618
2002	0.062	0.184	0.739	0.092	0.171	0.594	−0.072	0.167	0.667
2003	0.002	0.109	0.983	0.163	0.130	0.227	−0.162	0.126	0.213
2004	0.323	0.160	0.052	0.155	0.158	0.333	−0.150	0.149	0.322
2005	0.043	0.117	0.715	0.015	0.141	0.914	0.003	0.121	0.980

The statistically significant *P* value is noted in bold.

We did not consider sex‐linked genetic correlation because we did not find statistical support for sex linkage of any trait, and the estimated sex‐linked genetic variance for brightness was zero.

**Figure 1:**
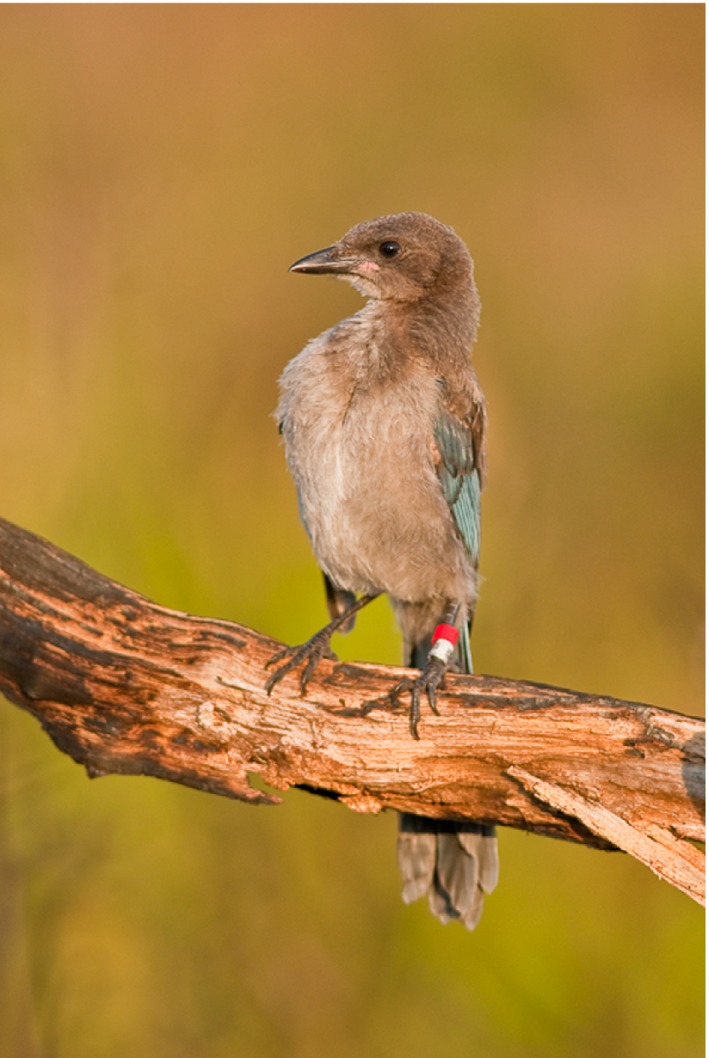
A juvenile Florida scrub‐jay. Although juveniles have blue wings and tails like adults, they are easily distinguished by their brown heads, which are blue in adults. Photograph by Reed Bowman.

We also examined the intersexual genetic correlation for brightness and UV chroma using bivariate models considering brightness or UV chroma in males and females as separate traits. Again, note that hue was not examined as the univariate models showed no genetic basis to this trait (Table 3). Because the same trait is not expressed in the same individual, the residual covariance and maternal covariance is not estimable and these were fixed to zero. The models did not converge when including the territory variance, and this term was therefore excluded, but as its effect was small (Table 3), this is unlikely to have a large effect on the interpretation of results, but they should nevertheless be made with caution (Fig. 1).

**Table 3:** Variance partitioning and the proportion of variance explained (PVE) for the autosomal and Z‐linked additive genetic basis of color traits in juvenile Florida scrub‐jays, where *V*_P_ is the phenotypic variance, *V*_A_ is the autosomal genetic variance, *V*_Z_ is the Z‐linked genetic variance, *V*_M_ is the variance due to maternal identity, and *V*_T_ is the variance due to territory identity. Because no additive genetic variance was detected for hue, Z‐linked variance was not estimated for this trait. In general, there was little support for Z‐linked variance of the different color traits in this species

Trait	Model	*V*_P_ (SE)	*V*_A_ (SE)	*V*_Z_ (SE)	*V*_M_ (SE)	*V*_T_ (SE)	PVE: $h_{A}^{2}$ (SE)	PVE: $h_{z}^{2}$ (SE)	PVE: *V*_M_ (SE)	PVE: *V*_T_ (SE)
Mean brightness	Autosomal	2.082 (0.131)	0.796 (0.181)	NA	0.531 (0.110)	0.717 E^−01^ (0.734 E^−01^)	0.382 (0.079)	NA	0.255 (0.046)	0.034 (0.035)
	Z‐linked	2.082 (0.131)	0.796 (0.181)	0.233 E^−06^ (0.374 E^−07^)	0.531 (0.110)	0.717 E^−01^ (0.734 E^−01^)	0.382 (0.079)	0	0.255 (0.046)	0.034 (0.035)
UV chroma	Autosomal	0.130 E^−02^ (0.815 E^−04^)	0.324 E^−03^(0.112 E^−03^)	NA	0.306 E^−03^ (0.727 E^−04^)	0.139 E^−03^ (0.615 E^−04)^	0.250 (0.084)	NA	0.236 (0.051)	0.107 (0.045)
	Z‐linked	0.132 E^−02^ (0.872 E^−04^)	0.255 E^−03^ (0.142 E^−03^)	0.749 E^−04^ (0.103 E^−03)^	0.310 E^−03^ (0.732 E‐^04^)	0.141 E^−03^ (0.616 E^−04^)	0.194 (0.109)	0.057 (0.077)	0.235 (0.055)	0.107 (0.047)
Hue	Autosomal	1946.9 (88.275)	0.119 E^−03^ (0.571 E^−05^)	NA	230.10 (60.237)	0.822 E^−04^ (0.395 E^−05^)	0	NA	0.118 (0.029)	0

We statistically tested variance components using likelihood ratio tests, which have a mixture of a chi‐squared distribution with one degree of freedom (testing a single variance component) and a chi‐squared distribution with null degrees of freedom, because of testing on the boundary of the parameter space (Self and Liang 1987), thus halving the *P* value for a standard chi‐squared test. To test the significance of the phenotypic and genetic correlations, we constrained the correlation to zero or one and compared the model to one in which the correlation was estimated. Note that because correlations (or more generally, covariances) need not be positive, these tests are chi‐squared distributed with 1 degree of freedom. All quantitative genetic models were run using the software ASReml 3.0 (Gilmour et al. 2009).

## Results

### Sexual dimorphism

Juvenile males and females differed significantly in mean brightness (*F*_1,1048_ = 26.443, *P* < 0.0001), UV chroma (*F*_1,1047_ = 43.732, *P* < 0.0001), and hue (*F*_1,1048_ = 18.354, *P* < 0.0001). Compared to females, males had plumage with lower mean brightness that is more UV‐shifted (Table 1). However, despite differing significantly in all components of plumage reflectance, SD was low, with ratios close to one (Table 1).

### Selection on trait coloration

**Figure 2:**
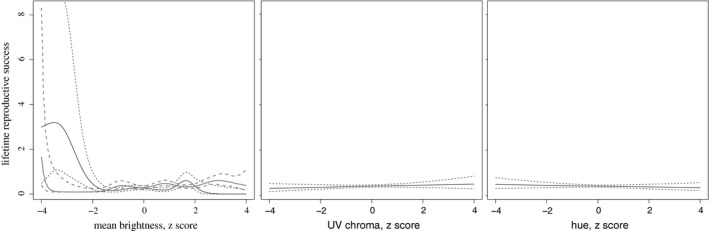
Predicted selection gradients ± standard error for mean brightness, UV chroma, and hue in Florida scrub‐jays. The figure for mean brightness shows females in black and males in gray. Sexes are shown together for UV chroma and hue because for these variables models that excluded sex had lower AIC values. We chose to visualize these gradients using curves rather than traditional linear Lande–Arnold selection gradients because the data are nonlinear.

We predicted that selection was acting on juvenile plumage, but did not detect a relationship between mean brightness (*β* = 0.03 ± 0.07, *P* = 0.66), UV chroma (*β* = 0.13 ± 0.07, *P* = 0.08), or hue (*β* = −0.10 ± 0.07, *P* = 0.53) on whether or not an individual bred. We also examined nonlinear patterns of selection on mean brightness, UV chroma, and hue between LRS and coloration using GAMs (Fig. 2). Mean brightness was not significantly related to LRS in males (*F*_1,1_ = 1.01, *P* = 0.32) or females (*F*_1.81,2.31_ = 1.06, *P* = 0.35). As a result, the selection gradients estimated from the GAMs were not significant for either sex (males: *β* = 0.17 ± 0.15, *P* = 0.24; females: *β* = −0.006 ± 0.25, *P* = 0.88). For UV chroma and hue, the models that excluded sex had the lower AIC values, so we present the results of these models here. No relationships between reproductive success and UV chroma (*F*_1.06,1.12_ = 0.29, *P* = 0.62) or hue (*F*_1,1_ = 0.13, *P* = 0.72) existed, nor were the selection gradients significant (UV chroma: *β* = 0.06 ± 0.12, *P* = 0.65; hue: *β* = −0.04 ± 0.11, *P* = 0.50). A linear selection analyses revealed significant (before correction for multiple tests) negative selection on mean brightness in 1993 (Table 2), but no other GLMs were significant. This is consistent with the pooled analysis of all years.

### Quantitative genetic basis of trait coloration

We found that both mean brightness (*h* ^2^ = 0.382 ± 0.079, *χ* ^2^ = 32.1, *P* < 0.001) and UV chroma (*h* ^2^ = 0.250 ± 0.084, *χ* ^2^ = 12.4, *P* < 0.001) were moderately heritable. For these traits, we also estimated sex‐specific variances and heritabilities. For mean brightness, the additive genetic variance and heritability estimates were 1.19 and 0.60 for females and 0.74 and 0.39 for males. For UV chroma, these estimates were 0.46 E^−3^ and 0.43 for females and 0.57 E^−3^ and 0.46 for males. Hue displayed no genetic variance (Table 3). Because the evolution of SD is often explained by sexual selection facilitated by sex‐linked genetic variance (Rice 1984), we looked for, but found no support for sex‐linked heritability, with estimates of heritability for mean brightness and for UV chroma (Table 3) that did not differ significantly from zero ($\chi_{1}^{2}$ = 0.38, *P* = 0.27). Sex linkage of hue was not examined because we found no genetic variance in this trait. Although small and nonsignificant, the addition of sex‐linked genetic variance decreased the estimated autosomal heritability from 0.250 to 0.194 (Table 3).

In addition to estimating additive genetic variance, we also estimated the influence of territory and nongenetic maternal effects on the color components. Territory explained a relatively small proportion of the variance in mean brightness and UV chroma and had no effect on hue (Table 3). Interestingly, maternal effects were relatively strong and common, explaining 10–25% of the variance of mean brightness, UV chroma, and hue (Table 3).

### Multivariate genetic basis of trait coloration

All color traits were significantly negatively correlated on the phenotypic level with correlations ranging from −0.084 between mean brightness and UV chroma to −0.536 between hue and UV chroma (Table 4). On the genetic level, we could only test the correlation between mean brightness and UV chroma because we did not find any indication of a genetic basis to hue (Table 3). We found support for a strong negative genetic correlation between UV chroma and mean brightness in this population, which was significantly different from zero (*r*_G_ = −0.821, $\chi_{1}^{2}$ = 516.92, *P* < 0.0001, Table 4).

**Table 4:** Phenotypic and genetic correlation estimates between color measures

	Mean brightness	UV chroma	Hue
Mean brightness	–	−0.084 (.031)**	−0.240 (.029)***
UV chroma	−0.821 (.177)***	–	−0.536 (.022)***
Hue	NA	NA	–

Phenotypic correlations (sex corrected) are above the diagonal and autosomal genetic correlations below with standard error in parentheses. Note that genetic correlations between hue and other traits were not estimable as no genetic variance was found for hue (see Table 3). Asterisks denote significance values against a correlation coefficient of zero: *P < 0.05, **P < 0.01, ***P < 0.001.

### Intersexual genetic correlations

The genetic correlation between the sexes was strong and not significantly different from unity for both mean brightness (*r*_G_ = 0.996, SE = 0.160, $\chi_{1}^{2}$ = 1.42, *P* = 0.12) and UV chroma (*r*_G_ = 0.948, SE = 0.190, $\chi_{1}^{2}$ = 1.62, *P* = 0.10) demonstrating strong evolutionary constraints for sex‐specific evolution of these traits.

## Discussion

We examined whether juvenile plumage coloration in Florida scrub‐jays was under selection and estimated the relative influence of genetic and environmental effects on plumage. Although plumage coloration is important in determining dominance relationships in this species, we found no evidence that any component of juvenile plumage reflectance was under selection.

Both mean brightness and UV chroma were moderately heritable, but without evidence of sex‐linked inheritance. A substantial part of the variation in UV coloration was due to maternal effects. Our heritability estimates are substantially higher than those for blue‐UV plumage color in blue tits (*Cyanistes caeruleus*) (Johnsen et al. 2003; Hadfield et al. 2007).

Plumage patterns are frequently sexually dimorphic (Hill and McGraw 2006), and SD might arise if the genes underlying these traits are sex‐linked (Rice 1984; Kirkpatrick and Hall 2004) or if the intersexual genetic correlation is low (Fisher 1931; Bonduriansky 2007; Williams and Carroll 2009). Despite early evidence of a sex‐linked sexually selected trait in guppies (Winge 1927), this has rarely been explored in other systems (but see, e.g., Ellers and Boggs 2002; Miura et al. 2011; Husby et al. 2013; Evans et al. 2014). In Florida scrub‐jays, the sexes are monomorphic to the human eye, but are dimorphic in the UV spectrum (Table 1). Despite theoretical work suggesting that the evolution of such dimorphism will be facilitated if genes for these traits are sex‐linked, we found no evidence of significant sex‐linked inheritance for any component of plumage color (Table 3). Moreover, the estimated sex‐linked heritability also was low ($h_{z}^{2}$ = 0.057). Although sex‐linked inheritance of sexually dimorphic plumage color has been documented in other species (e.g., Husby et al. 2013), it is unsurprising that we have found no evidence of it in the Florida scrub‐jay because SD is relatively minor (Table 1, Bridge et al. 2008). A similar finding has been reported in the barn owl (*Tyto alba*), where sexually dimorphic melanin‐based plumage traits are explained by polymorphisms in autosomal genes rather than by sex‐linked inheritance (Roulin and Jensen 2015).

Rice (1984) hypothesized that sexually antagonistic genes would accumulate on the sex chromosomes, but the optimum brightness and chroma of plumage is unlikely to differ between male and female scrub‐jays. Unlike coloration in guppies, where the advantage of being a brightly colored male is countered by the costs of higher predation risk (Godin and McDonough 2000), the UV‐shifted color associated with dominance in scrub‐jays is unlikely to carry such a high price; thus, the optima for both sexes should be similar. Additionally, SD is expected to increase with increasingly promiscuous mating systems (Dunn et al. 2001), and Florida scrub‐jays are socially and genetically monogamous (Woolfenden and Fitzpatrick 1984; Townsend et al. 2011).

Evolution of SD can be constrained if the intersexual genetic correlation is high (Lande 1980). We estimated the cross‐sex genetic correlation for brightness and UV chroma and found that these were high and not significantly different from unity. Such high intersexual genetic correlations combined with low sex‐linked genetic variance mean that the evolution of SD in these traits likely has been and is severely constrained.

Because UV chroma and hue signal social dominance in juvenile Florida scrub‐jays (Tringali and Bowman 2012) and dominant individuals gain priority access to resources (Drews 1993), we predicted that selection on these plumage traits would be evident. However, we detected no relationships between any metric of juvenile plumage color and fitness, measured either as whether or not an individual bred or its LRS. One possible explanation for this may be that juvenile dominance does not predict adult dominance and is therefore not important for gaining access to territories or mates. Alternatively, juvenile plumage coloration, which is molted prior to breeding, may not reflect adult plumage coloration. The observed environmental effects on UV chroma support this alternative. Environmental effects can cause variation in color across molts, leading to the apparent lack of selection on juvenile plumage. We observe a somewhat similar scenario in nestling and fledgling mass of this species. Although Mumme et al. (2015) found that nestling and fledgling mass are both important predictors of whether or not an individual breeds, suggesting a correlation should exist between plumage and probability of breeding, they do not predict LRS, indicating that while early development predicts who ultimately breeds, variation in LRS among breeders is explained by environmental effects (Mumme et al. 2015). Our results do not support the hypothesis that the observed juvenile SD is under sexual selection, and we think sexual selection is unlikely to act on juvenile traits. Studies are ongoing to determine whether juvenile plumage predicts adult plumage and whether adult plumage of Florida scrub‐jays is under selection.

Variation in territory identity explained very little of the variation in plumage reflectance, but maternal effects explained 10–25% of the phenotypic variance in all three reflectance variables. Although food limitation affects structural coloration (McGraw et al. 2002), more recently Peters et al. (2011) suggested that UV coloration might be more related to levels of the stress hormone corticosterone than to body condition. Corticosterone affects feather growth and development, with higher levels associated with weaker, lighter feathers containing more fault bars (DesRochers et al. 2009; Lattin et al. 2011). In Florida scrub‐jays, nest attendance behavior was highly variable among females and nestlings whose mothers spent more time further from the nest had higher levels of corticosterone (Rensel et al. 2010). Maternal effects on UV coloration may be mediated via nestling stress response to female incubation and provisioning behavior. The relationships of behavior and plumage coloration with corticosterone suggest that coloration and behavioral phenotype may be linked.

To understand the evolutionary dynamics of natural populations, we must know how selection and heritability affect traits of interest. We used a longitudinal study of a population of Florida scrub‐jays with pedigree and fitness data to study the evolution of sexually dimorphic plumage coloration in juveniles. We did not detect any association between plumage coloration and fitness even though juvenile plumage coloration is important in determining dominance relationships in this species. We found that both mean brightness and UV chroma were heritable, but no evidence of sex linkage for any plumage trait. The intersexual genetic correlation for brightness and UV chroma was near unity for both traits. Together, these results indicate that the evolution of sexual dimorphic plumage color is tightly constrained. Maternal effects explained much of the variation in plumage coloration, and it would be interesting to further explore the mechanism of this link.

## Conflict of Interest

None declared.

## Data Access

Data will be deposited in Dryad upon acceptance.
